# Protocol for diagnostic test accuracy study: the efficacy of screening for common dental diseases by Dental Care Professionals

**DOI:** 10.1186/1472-6831-13-45

**Published:** 2013-09-21

**Authors:** Richard Macey, Tanya Walsh, Anne-Marie Glenny, Helen Worthington, Martin Tickle, James Ashley, Paul Brocklehurst

**Affiliations:** 1 School of Dentistry, The University of Manchester, Oxford Road, Manchester, UK; 2 Woodlands Dental Practice, Birkenhead, UK

**Keywords:** Screening, Diagnostic test accuracy, Dental care professional, Sensitivity and specificity

## Abstract

**Background:**

The bulk of service delivery in dentistry is delivered by general dental practitioners, when a large proportion of patients who attend regularly are asymptomatic and do not require treatment. This represents a substantial and unnecessary cost, given that it is possible to delegate a range of tasks to dental care professionals, who are a less expensive resource. Screening for the common dental diseases by dental care professionals has the potential to release general dental practitioner’s time and increase the capacity to care for those who don't currently access services. The aim of this study is to compare the diagnostic test accuracy of dental care professionals when screening for dental caries and periodontal disease in asymptomatic adults aged eighteen years of age.

**Methods/design:**

Ten dental practices across the North-West of England will take part in a diagnostic test accuracy study with 200 consecutive patients in each practice. The dental care professionals will act as the index test and the general dental practitioner will act as the reference test. Consenting asymptomatic patients will enter the study and see either the dental care professionals or general dental practitioner first to remove order effects. Both sets of clinicians will make an assessment of dental caries and periodontal disease and enter their decisions on a record sheet for each participant. The primary outcome measure is the diagnostic test accuracy of the dental care professionals and sensitivity, specificity, positive predictive value and negative predictive values will be reported. A number of clinical factors will be assessed for confounding.

**Discussion:**

The results of this study will determine whether dental care professionals can screen for the two most prevalent oral diseases. This will inform the literature and is apposite given the recent policy change in the United Kingdom towards direct access.

## Background

The coalition Government is committed to delivering £15-20 billion in cost efficiency savings up to 2014 and they have recently announced a new contract for dentistry ([1,2]). Whilst looking to reduce expenditure and increase productivity, the National Health Service (NHS) remains committed to the vision of a healthcare service with quality as its organising principle ([1,3]).

Approximately 95% of the costs for NHS Dentistry are spent on routine care provided by General Dental Practitioners (GDPs); across the North-West alone, this amounts to £450 million per annum. However, just under half of the population do not attend the dentist and this group tends to be disadvantaged socio-economically and experiences the majority of the disease ([4,5]). In contrast, a large proportion of patients who regularly attend are asymptomatic do not require treatment [5], yet their care is delivered by the most expensive resource, the dentist. If unchallenged this situation where those with lowest needs consume the majority of resources is likely to deteriorate further, as the oral health of the population is improving [6].

Given that almost half of the patients who attend do not require any further treatment, the screening of asymptomatic regular attenders by a Dental Care Professional (DCP) could free up significant resources and increase the capacity to care.

In medicine a systematic review [7] found that services provided by Nurse Practitioners (NPs) were associated with higher levels of patient compliance and satisfaction [7]. In the United States, studies have suggested that Physician Assistants (PAs) can undertake 75% of the work of a physician, save resources and deliver high quality care for patients [8]. In 2003, the only systematic review undertaken on the use of role delegation in dentistry concluded that DCPs could screen for disease [9]. However, many of the studies were criticised for being of poor quality and few stemmed from the United Kingdom (UK) [9].

Savings could be delivered in NHS dentistry by simply extending the recall interval, as doubling this interval halves the cost. However, this does not account for the variation in risk of the disease across the practice population. This is important as research on dental caries suggests that once patients move from health to “disease active”, their risk of future disease is high [10,11]. As a result, it is important to maintain regular contact with patients to prevent dental disease from developing. In addition, the social acceptability of this extension in recall intervals to patients is likely to be poor. Hence, there is a challenge for NHS dentistry to provide preventive care, whilst delivering on quality, increasing productivity, releasing resources and increasing the capacity to care for those who currently can’t or don't access services [10].

Both the coalition Government and Steele’s review of NHS dentistry, argue for a shift from the current model of patient care. One alternative that has been suggested is the greater use of role substitution [12], where roles undertaken by the GDP are delegated to the dental team. In dentistry, DCPs are a heterogeneous group comprising of: therapists, hygiene-therapists, hygienists and extended duty dental nurses. Their roles are strictly defined by the Regulatory Body, the General Dental Council and they are *not permitted* to undertake an examination in order to formulate a diagnosis and treatment plan [13]. However, many patients do make use of services provided by DCPs between examination appointments with their GDP. Here, DCPs undertake the treatment prescribed by the GDP including preventive care and monitor the oral health of the patient in the intervening period. In addition, for some chronic conditions like periodontal disease, patients regularly see a DCP between visits to the GDP and effectively monitor and screen for disease.

### Study aim

The aim of this study is to determine the diagnostic test accuracy of DCPs when screening for common dental diseases in general dental practice.

### Objectives

To compare the diagnostic test accuracy of DCPs when screening for dental caries and periodontal disease in asymptomatic adults over eighteen years of age in general dental practice.

● The DCP screen will be classified as the index test.

● The GDP screen will be classified as the reference test.

● Sensitivity, specificity, positive predictive value, negative predictive value and diagnostic odds ratios will be reported (Table 1).

● Demographic (age, gender, post code) and clinical variables (number of teeth, dentures and restorations) will be tested for their impact on the ability to correctly screen for disease.

**Table 1:** Definitions of the features of diagnostic tests

Sensitivity	Probability that the test result is positive given that the condition is present
Specificity	Probability that the test is negative given that the condition is absent
Negative predictive value	Probability of the condition, given a negative result
Positive predictive value	Probability of the condition, given a positive test result

## Methods/design

### Study design

This is a Diagnostic Test Accuracy (DTA) study, the investigational methodology is designed based on the STARD checklist for the recommendations of studies of diagnostic accuracy [14]. All patients will be screened for dental caries and periodontal disease by a DCP (index test) and a GDP (reference test). Ethical approval was given by the NRES Committee North-West (Cheshire) on the 19th of February (13/NW/0010; 118638).

### Setting and study population

Dental practices will be sampled purposively, based on a list held of practicing DCPs from the North-Western Deanery. Patients over eighteen years of age attending dental practices for an asymptomatic examination (check-up) will be recruited and receive both the index test and reference test.

### Inclusion criteria for participating general dental practices

The inclusion criteria for the practices in the study will be:

● Conform to the General Dental Council’s “Standards in Practice”

● Registered with the Care Quality Commission

● Evidence of clinical governance (e.g. BDA Good Practice Scheme; Membership or Fellowship of the Faculty of General Dental Practice (UK))

● Sponsorship by a Consultant in Dental Public Health (no outstanding issues with their Area Team)

● Involved clinicians have a Professional Development Plan (to augment with research relevant training)

● Practice supported by a practice manager

● The employment of at least one dental hygienist or hygiene-therapist at the practice

● Large throughput of NHS patients, specifically practices with a minimum of three surgeries

### Inclusion and exclusion criteria for patients

The inclusion criteria for the patients in the study will be:

● an NHS patient

● a minimum of eighteen years of age

● asymptomatic and elective

● patient attending for a routine inspection (“check-up”)

● willingness to consent to study

The exclusion criteria for the patients in the study will be:

● patients in pain or requiring active intervention

### Participant recruitment

Patients due for a routine “check-up” will be contacted by the dental practice with an introductory letter and an information sheet explaining the aims of the study and what would be required of the patient. The patient will be asked to contact the dental practice to make an appointment on one of the dedicated study sessions. There will be contact details on the information sheet for the patient to seek any further clarification. A dedicated and trained member of the practice will then follow up this initial contact, through one of two means:

1. Telephone the patient directly to ascertain whether they are willing to take part in the study

2. Direct a patient enquiry when they telephone the dental practice to arrange their routine appointment

Upon arrival for their “check-up” the trained and dedicated member of the practice will remind the patient of the study and ask them if they are willing to take part. If the patient is still unsure then further details will be made available in the waiting room and the staff member will be available to address any questions.

If willing to consent, the patient’s eligibility will be confirmed and they will be asked to complete the consent form. Participants will be free to withdraw from the trial at any time, without explanation.

**Figure 1:**
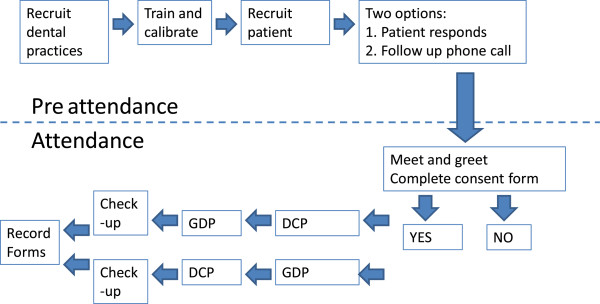
Recruitment process and transition of the patient through study.

The study population will be recruited consecutively (Figure 1), the data collection has been planned prospectively. A similar practice based study [15] reported a recruitment rate of 44% and since the demands placed on the patient are less onerous here, it is anticipated that the recruitment rate will be significantly higher.

### The reference standard and its rational

The reference standard should be the best possible method of determining whether the patient presents with the disease being assessed [16] and should be objective rather than subjective [14]. However, the clinical diagnostic test for dental caries is problematic [17]. Radiographs could be used, but this would be unethical and expose patients to unnecessary dose of radiation. In addition, there can be difficulties in achieving agreement of observer’s assessments [18]. To maintain a level of pragmatism and to allow a direct comparison to a DCP screen the reference test will be the GDP visual examination for caries and periodontal disease. All GDPs will be trained and calibrated to ensure they are maintaining high screening standards.

### Technical specifications and definitions

The diagnostic threshold for periodontal disease will be any pocket in a patient’s mouth that is within the black-band of a Basic Periodontal Examination (BPE) probe (3.5 mm to 5.5 mm); equivalent to a BPE score of 3 or above. Bleeding on probing will not be recorded given the potential for order effects. The threshold has been chosen as it represents the point where a full pocket charting is necessary and where further periodontal management is required over and above the Scope of Practice for the DCP. It is also in line with the recommendations of the British Society of Periodontology [19]. The caries assessment will be based on dried teeth and plaque may be removed to aid the determination of demineralization. Patients showing signs of frank cavitation, shadowing, opacity and evidence of caries that has reached the dentine should be classified as diseased.

The unit of analysis will be the patient. For both dental caries and periodontal disease, the DCP and the GDP will be asked to answer a hypothetical question on the record form based on their findings at a patient level: "Does the patient require any further investigation?" [20].

Clinicians will be asked to make their diagnostic judgement “as if” they were seeing the patient for the first time, rather than introducing any prior knowledge of the patient’s history into the judgement task. GDPs will be asked to avoid any assessment of risk and simply focus on whether the patient exceeds the diagnostic thresholds set for caries and periodontal disease.

### Patient Screening Process

A patient consenting to the study and meeting all other inclusion criteria will be allocated a unique patient identifier to ensure anonymity. A unique patient identifier reference sheet will be kept, which will enable the patients to be tracked and one patient record sheet provided to the DCP and one to the GDP.

To reduce order effects each practice will be randomly allocated using a permuted block sampling [21] system to either DCP first or GDP first. Over the course of the study equal proportions of DCP and GDP will perform the second screen.

The patient will see the DCP or GDP first (Figure 1). The first screen will be completed and then the patient will be directed to the second screen, which will be blind to the first screen. The DCP/GDP pairing will be the same for each participating practice. In order to avoid contamination the DCP and GDP examinations will be performed independently and the results will not be shared or compared by anyone other than the research team performing the statistical analysis. In this manner, a screening record of the patient's status in terms of caries and periodontal disease will be generated for the index and reference tests. The patient will not be informed of the results of the screen until they have completed the study and go on to have their routine “check-up”.

### Training and calibration of personnel

Both DCPs and GDPs will attend a compulsory training day, which will cover recruitment, consenting, screening process and patient record form completion. Stock photographs will be utilised to inform the screening for caries. Calibration will then be undertaken on a set of slides of carious and non-carious teeth [22]. Clinicians will be considered to have successfully completed their training when their values for sensitivity for the calibration exercise exceed 0.85; the threshold set by the World Health Organisation [23]. Levels for specificity will be set lower at 0.65, based on the results of the in-vitro study [24]. Clinicians that fail to reach this threshold will be asked to repeat the test before they can start the study. Kappa values for the agreement of each DCP/GDP pairing will be reported for this calibration exercise. Clinicians will also perform a test for periodontal disease to educate them of the correct force that should be applied whilst probing. This will be set to 25 grams, as recommended by the British Society of Periodontology [19].

### Mitigation of confounders

Certain clinical variables such as the number of teeth, dentures, restorations, crowns and implants present in a patient’s mouth have the potential to affect the accuracy of the screening process. As do factors such as pregnancy and smoking. To assess the impact of these potential confounders, both sets of clinicians will be asked to record basic information (Table 2). Restorations will be recorded at tooth level and categorised into three bands in line with the scoring of patients from the Adult Dental Health Survey 2009 [25], this is designed to highlight patients at the extremes of the restoration scale.

**Table 2:** Confounding factors

**Pregnant**	Yes □	No □		
**Smoker**	Yes □	No □	If yes, number per day	
**Dentures**	Yes □	No □	Full □ *exclude*	Partial: number of dentures 1 □ 2 □
**Total number of teeth**				
**Number of restorations and crowns**			None □	
			1 - 9 □	
			10 + □	

### Sample Size Calculation

Using the sample size methods devised by Flahault [26] where the prevalence of disease is less than 0.5 it is required that two assumptions are made. Firstly the expected sensitivity values of the new diagnostic test, secondly the minimum acceptable lower confidence limit. The in vitro study [24] testing the ability of different clinicians to assess photographs of potentially carious teeth showed a sensitivity across professional groups of 0.85. Given that clinicians found it more difficult to perform the screening test on photographs than patients it is expected that the sensitivity in the in vivo study will increase to 0.9. At the minimal acceptable lower confidence limit of 0.8 the number of cases with caries required is 235. Of the two diseases being studied caries has the lower prevalence of 20% [27]. This means 4x235 cases are needed without caries, giving a total of 1175 cases. The study will be conducted in ten dental practices with approximately 200 patients being seen for each GDP/DCP pairing.

### Statistical results and analysis

Results of the screening for caries and periodontal disease by DCPs (index test) will be compared to those of the GDPs (reference test) in all patients. The sensitivity, specificity, negative predictive value, positive predictive value and diagnostic odds ratios will be reported with 95% confidence intervals where appropriate (Table 1). The date of birth, gender, post code and clinical factors will be logged on the patient reference sheet and the resulting demographic characteristics of the study population reported. The time interval from the index test to the reference test will be reported and is expected to be less than twenty minutes. A cross tabulation of results of the results of index test by the results of the reference test will be reported, including indeterminate and missing results. The number of non-consenting patients will be reported as will any drop out during the study and the reason for any drop out will be reported. It is anticipated that once the patient has consented the drop-out rate will be minimal. It is not expected that any adverse events will be presented, however, this will be reported.

## Discussion

This study will be conducted in dental practices across the North West of England. The STARD diagram will be used in the reporting of results. This study aims to determine the diagnostic test accuracy of DCPs when screening for caries and periodontal disease. These results are intended to inform the potential for role-substitution in the general dental practice setting and provide evidence to contribute to the policy of direct access.

## Competing interests

The authors declare that they have no competing interests.

## Authors’ contributions

All authors made substantial contributions to conception and design of the study. RM, PB and JA were responsible for drafting the protocol. RM, PB and AMG are responsible for overall design and management the trial. TW and HW contribute to the statistical and data management. MT, JA and PB are responsible for clinical design of the study. All authors read and approved the final manuscript.

## Pre-publication history

The pre-publication history for this paper can be accessed here:

http://www.biomedcentral.com/1472-6831/13/45/prepub
